# Perceptions and Experiences of School Teachers During the Implementation of a School-Based Deworming Activity in Kenya

**DOI:** 10.24248/EAHRJ-D-18-00028

**Published:** 2019-07-30

**Authors:** Doris W Njomo, Cynthia Kairu, Janet Masaku, Faith Mwende, Gladys Odhiambo, Rosemary Musuva, Elizabeth Matey, Isaac G Thuita, Jimmy H Kihara

**Affiliations:** a Eastern and Southern African Centre for International Parasite Control, Kenya Medical Research Institute, Nairobi, Kenya; b Centre for Global Health Research, Kenya Medical Research Institute, Kisumu, Kenya; c Centre for Microbiology Research, Kenya Medical Research Institute, Nairobi, Kenya; d Ministry of Education, Science and Technology, Nairobi, Kenya; e Division of Vector-Borne Diseases, Ministry of Health, Nairobi, Kenya

## Abstract

**Background:**

Primary school teachers are key stakeholders in the success of school-based deworming activity as they are responsible for drug administration and provision of health education to the School-Age Children (SAC). In Kenya, the National School-Based Deworming Programme (NSBDP) for control of soil-transmitted helminths and schistosomiasis was initiated in the year 2012 in prioritised areas. By the year 2013, over 6 million SAC had been treated. The present study sought to assess the teachers' perceptions and experiences of the school-based deworming activity in an effort to improve programme effectiveness.

**Methods:**

Qualitative data were collected, using in-depth interviews, in 4 subcounties of the coastal region of Kenya. Using purposive selection, 1 primary school teacher from each of the 38 schools also purposively selected participated in the study. The data were audio-recorded, transcribed, coded and analysed manually by study themes which included: reason for being selected for training to administer drugs; perceptions of training content and duration; experiences during drug acquisition, administration and record-keeping and motivation to continue participating in the deworming of school-age children.

**Results:**

Half of the teachers indicated that they were selected to administer drugs to children as they were responsible for school health matters. The duration and content of the training were considered sufficient, and no challenges were faced during drug acquisition. Challenges faced during drug administration included non-compliance and experience of side effects of the drugs. No major problems were experienced in record-keeping, although the teachers felt that the forms needed to be simplified. Improvement of the children's health and class performance was reported as a source of motivation to the teachers to continue administering the drugs. Fellow teachers were reported to have given moral support while over half of the respondents indicated that parents did not provide much support.

**Conclusion:**

Generally, teachers have positive experiences and perceptions of the deworming activity. There is, however, a need to involve all stakeholders especially the parents through the school board of management to help counter non-compliance and possibly support in providing meals to the children to help minimise side effects after drug consumption. Inadequate moral support and incentives are negative factors on the teachers' motivation.

## INTRODUCTION

Soil-transmitted helminthiases (STH) are a group of parasitic diseases and are among the Neglected Tropical Diseases (NTDs) that cause huge disease burden in the developing world.^1^ Neglected tropical diseases (NTDs) refers to a group of mainly chronic, debilitating and often stigmatising diseases,^2^ that generally persist under conditions of poverty or impoverished populations, the most affected being concentrated almost exclusively in Low and Middle Income (LMIC) countries.^3^ More than 2 billion people are estimated to be infected with STH worldwide, with disease burden being caused primarily by 3 main species of STH; *Ascaris lumbricoides* (roundworms), *Trichuris trichiura* (whipworm) and *Necator americanus* and* Ancylostoma duodenale* (hook-worms).^4^

School-age children (SAC) and pre-school age children (PSAC) are the most vulnerable groups that harbour the greatest number of worms, parasites *A. lumbricoides* and* T. trichiura* being particularly the most prevalent and intense.^5^ It is estimated that over 400 million SAC are infected with 1 or more major species of worms,^6^ with 44% of the infection being concentrated in Nigeria, Democratic Republic of Congo, South Africa and Tanzania making children them main targets for treatment.^7^ In Kenya, surveys carried out in 2008 found that an estimated 5 million (56.8%) school-going children were infected with parasitic worms (including STH and schistosomiasis) and required mass deworming.^8^

Morbidity of the disease is often linked to the intensity of worm burden rather than the mortality it causes, hence children with a heavy STH intensity suffer health problems, such as general malaise, diarrhoea, abdominal pain, malnutrition, and intestinal blood loss, which is accompanied by iron deficiency in cases of hookworm infection.^5^ Impairments in physical and cognitive development are also associated with chronic STH infections leading to poor school performance and absenteeism in children.^9^ For the control of STH, the World Health Organization recommends the periodic mass administration of anthelminthic drugs albendazole or mebendazole, ie, preventive chemotherapy, which includes its integration in the existing school-based systems.^4,10^ Treatment of school-aged children in schools is especially favoured because it is among the most cost-effective approaches in minimising infection intensity by utilising school infrastructure and reducing distribution costs.^5^ Furthermore, school-based control programmes can also extend to other high-risk groups such as; children not enrolled in schools, women of childbearing age and adults at high risk in certain occupations.^7^

Surveys demonstrate that coastal and western regions of Kenya have the highest, 32.2% prevalence of STH in school-age children. Hookworm infections are also particularly high in south-western Kenya and in Kilifi and Kwale counties in the coastal region.^11,12^ To counter the negative impact of the worms on children's health and education, the National School-based Deworming Programme (NSBDP) was launched in Kenya in the year 2012. The programme targets all school-age children in subcounties of high densities of STH infection located in Western, Nyanza, Coast and parts of Rift-valley regions.^13,14^

School-based control programmes do not often comprise deworming, improvement of water and sanitation and health education, although this would be ideal. Studies have shown that the successful combination of these strategies in helminth control programmes can reduce the transmission of schistosomiasis and STH.^15^ Deworming programmes rely on the use of existing school infrastructure, especially primary school teachers to administer deworming tablets to their pupils.^13^ Thousands of teachers in endemic countries take a leading role in administering deworming drugs to SAC and PSAC and providing health education to parents and pupils in their respective schools. In Kenya, a total of 16,000 teachers were trained in the first phase, during the year 2012 of the NSBDP.^8^ Training takes place through a cascade where National level Ministry of Health and Ministry of Education personnel train officers at the County level who then train those at the subcounty level who are then charged with the responsibility of training the teachers to administer the drugs to the school-age children. The primary school teachers are expected to ensure that all PSAC in ECD Centres within the primary school compound and outside receive the drugs.

Several helminth control programmes have demonstrated that recruiting and training teachers for the implementation of school-based deworming programmes is advantageous. Teachers are generally trusted by children and their families, hence are usually more willing to accept health interventions,^15^ thus playing an important role in benefiting the overall health and educational outcomes of children.^14^ Studies show that there is a need to better understand teachers' knowledge, skills and attitudes as health promoters in any school health programme to promote their motivation and build their capabilities to implement health promotion in schools.^16^ In the programme, the Health personnel specifically, the community health extension workers (CHEWs) are charged with the role of community sensitisation for awareness creation and mobilisation and provision of technical support to the teachers during the deworming day especially in the management of the occurrence of any side effects.

Through the School-based Deworming Programme (SBDP), surveys conducted in Kenya show that, by 2013, over 6 million children had been dewormed.^14^ The results of a survey conducted prior to deworming in 2012 showed that the prevalence of STH in PSAC in Matuga and Msambweni sub-counties in Kwale County was 27.8% and 66.7% respectively while that of Malindi subcounty in Kilifi County was 44.5%.^12^ Most recent surveys show that after 3 years of programme implementation, the overall prevalence of STH infection has reduced to 16.4% from a prevalence of 32.3% at baseline.^12^ This paper is part of a larger study entitled “Evaluating Different Drug Delivery Approaches for Treatment of Soil-transmitted Helminthiasis and Schistosomiasis Infections in the NSBDP among children attending Early Childhood Development (ECD) Centres in Coastal Kenya” which was carried out after mass drug administration (MDA) of 2014. The present study assessed the primary school teachers' experiences and perceptions of; teacher selection and training, community sensitisation, drug acquisition, record maintenance, teachers' motivation and incentivisation during drug administration to children. The study sought to identify areas for improvement or continuity in the implementation of the school-based de-worming programme.

## METHODS

### Study Site

The study was conducted in 4 endemic subcounties: Matuga, Msambweni, Lunga Lunga and Malindi in Coastal Kenya. These subcounties were selected on the basis of a high prevalence of infection and the distance between some primary schools and pre-schools being ≥2 km. Three of the subcoun-ties (Matuga, Msambweni, and Lunga Lunga) are located in Kwale County, whereas Malindi is located in Kilifi County.^17^ Kwale County covers an area of 8270 km^2^ and lies between Latitudes 30 3‘and 40 45‘South and Longitudes 380 31‘and 390 31‘East.^17^ The total population was projected to be 759,318 in 2015,^18^ with the majority of its population living in poverty. Kwale County experiences monsoon type of climate marked by hot and dry weather from January to April/May and cool temperatures from June-August.^17^

Health-care service delivery in Kwale is generally poor due to inadequate health workers and health facilities and shortage of medicines, medical supplies and equipment. The county has a total of 3 government hospitals, 8 health-care centres, 64 dispensaries including 2 private hospitals. The patient to doctor and nurse ratio stands at 1: 76,741 and 1: 3,133 respectively which is far below the WHO prescribed ratio of 1:1000.^19^ Access to health facilities is also a major challenge as the average distance to the nearest health facility within the county is 7 km.^18^ The most common diseases reported are; malaria, diarrhoea, flu, respiratory diseases and stomach ache. There is also a high prevalence of HIV/AIDS.^18^

Malindi subcounty is located 120 km east of Mombasa and covers an area of 7,750 km^2^ and has a population of 424,081.^20^ This population is served by 3 hospitals, 4 private chemists and 24 dispensaries. A major challenge for Malindi residents is the accessibility to health facilities; statistics show that in rural areas, the nearest health facility is 3 kilometres away. Aside from long distances, high poverty levels also make health care inaccessible to the people.^21^ Malaria, respiratory tract infections, skin infections, diarrheal diseases and intestinal worms are the most highly reported diseases that cause morbidity.^21^

### Study Design and Setting

This was a qualitative study that utilised in-depth interview method for data collection. The data were collected in May 2014, after the February 2014 round of deworming. The data collection instrument was developed by trained KEMRI researchers through a consultative process with the programme implementers. The questions were then translated into Kiswahili by a hired independent translator and then back-translated into English by a different translator to confirm that the intended meaning of the questions had not changed. The interviews were conducted at the primary schools. All primary schools that serve stand-alone pre-schools that are ≥2 km away were identified with the help of the Early Childhood Development Education) Office and considered for the study.

### Study Population and Data Collection

A total of 38 primary school teachers from 38 primary schools with pre-schools attached to them who administered anti-helminths to the school children during the February 2014 round were selected purposively and in-depth interviews conducted by trained KEMRI researchers to gather information on their perceptions of and experiences during the deworming activity. In the selection, researchers mini-mised bias by ensuring that head teachers and health teachers who are charged with the responsibility of administering treatment to the SAC and PSAC were recruited for the study. The number of interviews was determined by the level of saturation, ie, once no new information was being received, then no more interviews were conducted. The design was iterative, and there was a back and forth process which included data collection and analysis and further sample selection, therefore, giving early insights and influencing the selection of more participants up to the point where no more new information was being gathered. Standard procedures including maintaining a neutral stance, probing and allowing the respondents to express themselves without asking leading questions, asking general questions before specific questions and varying questions wording to avoid seeming repetitive were adhered to while conducting the IDIs.^22^ Each interview took a minimum of 40 minutes to a maximum of 50 minutes at a venue conducive to the process. Data were recorded using audio-cassettes in *Kiswahili* the local language commonly used in the coastal region. The data were then transcribed and translated and back-translated into English by trained KEMRI investigators. Double transcription and translation and back-translation was done among the investigators to agree on the meaning of the transcripts and minimise bias.

### Data Management and Analysis

The data that were captured through note-taking was compared with that captured through voice-recording to ensure that all important information was captured. The analysis was done manually according to study themes which were determined before the analyses. A code sheet was created following the in-depth interview guides after which, the textual data were coded into selected themes, and a master sheet analysis was carried out, giving all the responses from the IDIs a theme. Thematic analysis was used where responses were categorised into themes and then ideas formulated by looking at the patterns of responses. Analysed data were presented in text form. The identified themes included the reason for being selected for training to administer drugs; perceptions of training content and duration; experiences during community sensitisation, drug acquisition, drug administration and record-keeping and motivation to continue participating in the deworming of SAC and PSAC. The data analysis was approached deductively, wherein general statements were used to form specific conclusions. The quantitative data from the socio-demographic profiles, ie, age, highest level of education, religion, and marital status was managed using Excel (Microsoft Corp., Redmond, WA, USA) spreadsheets.

### Ethical Considerations

Ethical clearance was received from the Kenya Medical Research Institute (KEMRI)/National Ethical Review Board (Protocol Number 2547) and written informed consent sought from all the study participants. All the participants were adults above the age of 18 years, and therefore, no parents/guardians were expected to give consent on behalf of a minor. All information given by the study participants was kept confidential, and anonymity was highly observed as no personal identifiers were used during data entry, analysis and presentation. Confidentiality was ensured through coding the transcripts by giving a number to each respondent and a name based on the subcounty, ie, LLG for Lunga Lunga, MSB for Msambweni, MLD for Malindi and MAT for Matuga. PT was used to distinguish the Primary Teachers from other study participants of the larger study.

## RESULTS

A total of 38 teachers from 38 primary schools consented and participated in the full in-depth interviews, 28 males and 10 females. The oldest teacher interviewed was 58 years old and the youngest 23. Regarding religion, 23 teachers were Christians, and 15 were Muslims; 34 were married, and 4 were single. All teachers had received college-level training. From the total of 38, 12 were from Malindi, 8 from Msambweni, 10 from Matuga and 8 from Lunga Lunga subcounty (Table).

**TABLE:** Background Characteristics of the In-Depth Interview Participants

Variable		Male	Female
**Number of participants**		28	10
**Age**	Median (range)	47 (23-58)	47 (29-53)
**Marital status**	Single	3	1
	Married	25	9
**Religion**	Christian	15	8
	Islam	13	2

### Teachers' Selection

With regards to the criteria used to select teachers to administer the drugs during the NSBDP, half (n=19) of respondents indicated that they were selected because of their role in the school as the health teacher. About a quarter of the participants (n=10) indicated that they were selected because it is their responsibility as head teachers to oversee the exercise. Only a small minority (n=3), indicated they were selected because they had in previous rounds dewormed the school children. A health teacher stated that:

**I am the school health teacher, and they needed the school health teacher and either the deputy or the head teacher so being the school health teacher, I was the right person to go.”**

(MLD PT004)

### Training

A majority (n=31) of respondents indicated the training on drug administration took 1 day. On the content of training, a large majority (n=31) indicated they were trained on how to administer drugs and manage side effects, and educated on intestinal worms and their effect on health. A teacher stated:

**We were trained on how to administer, the drugs the side effect of the drugs and how to prepare the children to be ready for the drugs and also discussed the worms different types and how they affect the learners and why they were targeting the schools they said because they target the young children who can only be found in school, and that is why they did not go to the villages.**

(MSB PT 007)

Only a small minority (n=5) mentioned they were trained on how to maintain records during drug administration. Less than half (n=15) of respondents indicated the training was satisfactory. A majority of the teachers indicated that they would have liked to have more training on the worms, transmission cycle, and preventive measures and probably the length of training should be increased for them to learn and understand more about the record-keeping. A teacher stated:

**I had challenges, especially in the writing of the summary; it was a bit technical…**

(MAT PT 009)

### Community Sensitisation

When teachers were asked on the methods used to inform the community about the school-based deworming activity, a majority (n=28) indicated they used the school children to inform community members about the exercise. Less than a third (n=11) stated they used posters with another 11 indicating that they used other school teachers and school committee members. More than half (n=21) of respondents stated that the methods they used to sensitise the communities were adequate to reach the parents. However, about a half (n=18) indicated that using children alone to sensitise the community members were not adequate. A head teacher stated:

**That method that we ourselves used was not adequate because you cannot rely 100% with the children since some of them might not tell their parents about the exercise.**

(MAT 004)

To improve the means of community sensitisation, about a third (n=13) proposed that using media sources such as; radio, public address systems and posters would be the most effective means of sensitising community members. A teacher thus stated:

**Maybe posters, we put them at the market centres and these days we have got these “FM” radios, local example here we have got “Kaya FM” which Kwale residents listen to that can also assist.”**

(MAT PT 002)

A teacher further stated:

**I think more still needs to be done like if it is done using these public communication devices, … like we have a vehicle perhaps going across the region announcing what was going to happen and perhaps educating briefly the community members on why it is happening, I think it would bring more attention to the people and the attendance would improve.**

(MLD PT011)

Other suggestions given by slightly more than a quarter (n=10) of respondents to improve on community sensitisation were to use local leaders such as village elders and chiefs.

### Drug Acquisition

With regards to drug acquisition, more than half (n=25) of the respondents indicated they did not face any problems during the collection of drugs which was done after the teacher training session. A large majority (n=34) of respondents indicated they received the drugs on time, and all 38 reported they received sufficient amount of drugs to carry out the treatment with some indicating that they received excess drugs. A health teacher thus stated:

**There was no problem because we were given the drugs there and then after the training and we came with them to the school and stored them waiting for the deworming day. The quantities were more than enough and some of them we took back to the Zonal Education office.**

(LLG PT 002)

### Mode of Drug Administration

More than half (n=22) of the teachers indicated that they faced challenges during drug administration. The greatest challenge reported was children's refusal to take drugs out of fear of their safety. The teachers reported that children, however, eventually complied with treatment after they swallowed the drugs themselves to demonstrate their safety. There were isolated reports of children refusing to comply with treatment because of religious beliefs. A health teacher stated that:

**It was somehow challenging because there are some religious sects which refused their children who are in that denomination to take drugs. They refused totally; for example, those from Imani Mwenga and One Faith Schools, they refused completely and ended up running away from schools.**

(MLD PT005)

Other challenges experienced by teachers during drug administration included; late arrival, poor attendance and failure to have eaten a meal before drug consumption. A head teacher stated that:

**Challenges were there; for instance, we were told that the children should have eaten at their homes before taking the drugs. Knowing whether or not a child has eaten is a problem. Another one is about attendance, as I explained to you. You find that some pupils do not like medicine; once they hear that there shall be de-worming on a certain day, they do not come to school.**

(LLG PT004)

Less than half (n=15) of the respondents, however, indicated they did not face any challenges during drug administration.

Regarding side effects after drug consumption, more than half (n=22) of the respondents reported that children did not experience any side effects after consuming the drugs. However, less than half (n=14) reported that children experienced side effects which were transient such as; dizziness, nausea, vomiting, weakness and stomach pain, especially after consumption of praziquantel for schistosomiasis. The main strategy used by teachers to manage side effects was directing children to rest in a cool shady area. A teacher thus stated:

**… there was dizziness, the children were complaining of headache, and feeling dizzy, it was more the ones who took bilharzia drugs, those children who were feeling dizzy, feeling like vomiting, they would just rest, but after some time in the afternoon, it was fine.**

(MSB PT 002)

Another teacher also stated that:

**What I can talk about is the way this drug is very strong. If there could be the provision of a feeding program, which would be nice so that the children can have the energy to receive the drug well. It is sad that they receive the drugs on an empty stomach.**

(LLG PT 04)

### Record Maintenance

A large majority (n=29) of the respondents indicated that they had enough forms to maintain records after drug administration. Only 3 teachers indicated that the forms were not enough.

A majority (n=28) of respondents reported that they did not face major challenges in recording keeping, while 9 teachers reported having faced challenges in filling the forms as in their opinion, they were quite technical. One teacher thus stated:

**…about how to fill in the totals at the bottom. You see when you got 1 form, it was easy, but when you got more than 1 form, it was a bit tricky where to write the final summary, it was bit challenging…it was a bit technical.**

(MLD PT 006)

### Motivation

Out of the 38 primary school teachers interviewed, more than half (n=23) indicated that improving the children's health and performance in class motivated them to continue administering the drugs to the children. A head teacher stated:

**… the motivation is that these children are ours and if they have some good health they will perform well, and if they perform well, it is also good.**

(MSB PT 03)

### Support Received

A majority (n=29) of primary school teachers reported that they received support mostly from fellow teachers in the facilitation of the deworming exercise and record keeping. However, slightly more than half of respondents (n=20) reported they did not receive enough support, especially from parents who failed to participate and bring their children to receive the drugs. The failure of the parents to take their children to schools to receive the treatment may have been as a result of the inadequacy in community sensitisation. Another teacher stated that:

**The least supportive now, I can say, were the parents.**

(MLD PT010)

Out of the 38 respondents, only 11 indicated they received financial support for transport, lunch and communication allowance. A small minority (n=7) however indicated that in future, more financial support due to the heavy workload would make the teachers feel appreciated. A head teacher stated that:

**I don't think I have anything to add maybe just that next time, you should consider the teachers, even if its fifty shillings each, just as a token of appreciation. So that they can feel appreciated…**

(LLG PT 006)

## DISCUSSION

Results reported in this study show that generally, the standard of activities being implemented during the NSBDP is satisfactory based on the results showing high treatment coverage of targeted schools; 94% and 98.1% of Kilifi and Kwale counties, respectively.^12^ but a few areas need to be addressed for an improved programme. The teachers who administered drugs to children were selected mainly because of their role in the school as health teachers. Furthermore, some teachers who attended the training were selected because it is their responsibility as the school head teacher. This follows the Kenya National School-based deworming programme implementation model which requires for particularly, primary school heads and health teachers to receive training on school-based deworming by Division-level trained personnel.^23^

According to the results of this study, the duration of training was 1 full day. In terms of the content covered during the training, most teachers mentioned they were trained on how to administer drugs, manage side effects and educated about intestinal worm transmission, effects and prevention measures. A study conducted to assess the implementation of the NSBDP in Bihar State, India shows similar results.^24^ Other studies have reported that the effective training of teachers contributes to increasing the acceptance of community members to participate in deworming exercises, by demystifying rumoured myths or misconceptions.^13,25^ How ever, the present study results also show that a small number of teachers indicated that they received training on record-keeping, which is contrary to stipulations of the NSBDP module, which highlights training on filling and managing of monitoring forms as very important in teacher training sessions.^13^ The programme implementers are thus advised to ensure that the training given on record-keeping is adequate for accurate data to be transmitted to the Health and Education authorities.

Using students to inform parents and other community members about school-based deworming activities was the most common means of awareness creation due to its convenience. There was, however, a feeling among teachers of the need to improve the means used to sensitise the community, to ensure that the parents are properly informed about the deworming activities. Reports indicate that awareness creation and community sensitisation efforts are crucial for achieving high coverage of treatment as it aims to motivate parents to bring their children for treatment. Programme implementers are advised to combine several approaches to maximise on compliance taking the community leaders as important in reaching more community members. The Ministry of Health and Welfare in India recommended using a mix of media sources that are culture-specific or most effective in the local context, such as print media, posters and newspapers, radio, public announcements, or television.^26^ Odhiambo et al^27^ reported that more involvement of local community leaders who are respected in the community is also encouraged as they play a pivotal role in sensitising the community and can lead to increased ownership and acceptability of the programmes. Similarly, results of the present study demonstrate that teachers highly recommend the use of media sources (posters, radio, public announcements and television) and local leaders through village meetings (*barazas*) to sensitise community members on deworming activities.

The teachers faced no challenges at the time of drug acquisition, and the amount of drugs received was sufficient. Lack of challenges in drug acquisition and receiving sufficient quantities are important factors in ensuring that all targeted children receive the treatment during the deworming day. These results are contrary to those of a study on lymphatic filariasis elimination carried out in Kenya,^28^ and different states and districts in India^29,30^ which reported a delay in supplies as well as inadequate amounts of drugs being supplied to the drug administrators. The study conducted in India by Babu et al^30^ associated timely and adequate drug supply with promoting high coverage and compliance to drugs, hence contributing to the smooth and effective implementation of deworming programmes.

The results of this study demonstrate that a number of challenges were faced by teachers during the drug administration activity. One of the reported challenges was the children's refusal to take the drugs. This, however, was resolved when teachers took drugs in front of the children to demonstrate their safety, which led to the children's compliance. A study carried out in Turkey demonstrated that children are more willing to comply with treatment if it is delivered by a trusted source such as a parent or teacher.^31^ Religious beliefs were also found to be a reason for children's refusal of treatment, although the cases were few. A study conducted in western Kenya also reported cases of community members resisting treatment for schistosomiasis due to religious beliefs.^28^ Religious and sociocultural barriers are major hindrances to health-seeking, especially among rural communities. Increased sensitisation and education of spiritual leaders to advocate for compliance with health interventions have been recommended as a strategy to overcome such religious and other sociocultural barriers.^32^

The present study also reported side effects cases following treatment with praziquantel. Signs presented were considered mild and ranged from; dizziness, nausea, vomiting, weakness and stomach pain, which were effectively managed by teachers. The teachers associated the occurrence of side effects with the failure to feed children before treatment. The health authorities are called upon to ensure that there is supervision of the deworming activity in schools, so that side effects are managed in good time to counter any chances of negative publicity. Similarly, a study in Uganda reported that side effects occurred especially after taking praziquantel on an empty stomach.^33^ A number of studies have recommended the taking of deworming drugs with food to mitigate side effects.^34,35^

According to the results of this study, majority of teachers had minimal challenges in filling of monitoring forms during drug administration which can be attributed to success of the programme that has shown a reduction in prevalence and intensity of STH.^12^ Efficacy in the distribution of programme support materials such as monitoring forms have been reported to potentially determine the success in achieving smooth implementation of deworming programmes. Furthermore, information gathered from forms filled out by teachers is crucial as it is used to evaluate programme success alongside the target population, making the filling of forms an important part of the implementation process.^36^ It is, however, important for the programme managers to note the negative reports by teachers that emphasised on the complexity and technicality of the monitoring forms, which could negatively affect programme effectiveness.

Results of the present study have highlighted the improvement of the health and general wellbeing of the school children as the major motivating factor for teachers to administer drugs. Furthermore, teachers were increasingly motivated because improvements in children's health were directly associated with reduced absenteeism and increased concentration in class and thus improved class performance. Primary school teachers play key roles in the success of the NSBDP, and the programme implementers are encouraged to ensure that teachers feel appreciated. These results corroborate with studies carried out in Kenya and Tanzania,^37,38^ which stated that community volunteer drug distributors were more likely to be motivated to administer drugs due to intrinsic reasons such as; helping the community and improving their health.

Community support, according to the present study results, has been identified as an important incentive in increasing teachers' motivation. The importance of peer support in facilitation of the deworming exercise and recording keeping was especially highlighted by the teachers. This may have contributed to building teachers morale to participate in the deworming exercise, similar to the results of the study carried out in Kenya.^37^ The present study, however, demonstrated that teachers somehow felt demotivated by lack of active participation of the parents during the deworming exercise. The Programme implementers are thus required to implement health promotion campaigns through intersectoral approaches and involve the parents in the control strategies for buy-in and support. Results of other studies have shown that the support by community members, such as parents, is critical for making drug administrators feel appreciated in the communities to which they provide services^39^ and is also crucial to the success of the programme.^15^

### Limitations

The study's limitation is recall bias on questions pertaining to the training as the teachers in the far-flung subcounty of Lunga Lunga were interviewed at a time exceeding 3 months from the time that they had received the training although within 3 months of administering treatment to the school-age children.

## CONCLUSION

The teachers generally have positive experiences and perceptions of the school-based deworming activity. The study has, however, identified critical factors that are important for the improvement of the NSBDP from the teachers' perspective. There is a need to involve all stakeholders especially the parents through the school board of management to help counter non-compliance and possibly support in providing meals to the children to minimise side effects after drug consumption. Intersectoral approaches are recommended for improved awareness creation. The treatment record-keeping forms may need to be simplified for the teachers to make use of them easily. Inadequate moral support and incentives are negative factors on the teachers' motivation and there is need for all stakeholders, particularly parents, to get fully involved in the programme.
